# Micronutrient Status and Breast Cancer: A Narrative Review

**DOI:** 10.3390/ijms25094968

**Published:** 2024-05-02

**Authors:** Alicja Forma, Arkadiusz Grunwald, Patryk Zembala, Jacek Januszewski, Adam Brachet, Roksana Zembala, Kamila Świątek, Jacek Baj

**Affiliations:** 1 Chair and Department of Forensic Medicine, Medical University of Lublin, Jaczewskiego 8b, 20-090 Lublin, Poland; arek.grunwald.2@wp.pl (A.G.); adambrachet@gmail.com (A.B.); 2 Faculty of Medicine, Medical University of Warsaw, Banacha 1A, 02-097 Warsaw, Poland; patrykx0@hotmail.com; 3 Department of Correct, Clinical and Imaging Anatomy, Chair of Fundamental Sciences, Medical University of Lublin, Jaczewskiego 4, 20-090 Lublin, Poland; jacek.januszewski000@gmail.com (J.J.); kk.swiatek21@gmail.com (K.Ś.); jacek.baj@umlub.pl (J.B.); 4 Faculty of Medicine, Cardinal Stefan Wyszynski University in Warsaw, Wóycickiego 1/3, 01-938 Warsaw, Poland; roksana.zembala@gmail.com

**Keywords:** micronutrients, breast cancer, vitamins, metallomics, nutritional status, nutrition, trace element

## Abstract

Breast cancer is one of the most common cancers worldwide, at the same time being one of the most prevalent causes of women’s death. Many factors such as alcohol, weight fluctuations, or hormonal replacement therapy can potentially contribute to breast cancer development and progression. Another important factor in breast cancer onset includes micronutrient status. In this narrative review, we analyzed 23 micronutrients and their possible influence on breast cancer onset and progression. Further, the aim of this study was to investigate the impact of micronutrient status on the prevention of breast cancer and its possible influence on various therapeutic pathways. We researched meta-analyses, systemic and narrative reviews, retrospective studies, as well as original studies on human and animal models. The results of these studies indicate a possible correlation between the different levels of micronutrients and a decreased risk of breast cancer as well as a better survival rate. However, further studies are necessary to establish adequate doses of supplementation of the chosen micronutrients and the exact mechanisms of micronutrient impact on breast cancer therapy.

## 1. Introduction

Despite the introduction of various novel therapeutic strategies as well as the discovery of new diagnostic biomarkers that could facilitate earlier breast cancer diagnosis, breast cancer still remains one of the most common cancers worldwide and the fourth most common cause of women’s death [1]. A vast majority of breast cancer incidence occurs in women while breast cancer in men is very rare—less than 1% of all breast cancer cases occur in men [2].

Generally, the most common non-modifiable risk factors for breast cancer include female sex, older age, family history of breast cancer, specific genetic mutations, greater breast tissue density, or previous radiation therapy. Women have a greater chance for this cancer to occur because it is also mostly associated with early menarche (before the age of 12), late natural menopause (after the age of 55), not having children, or first pregnancy over the age of 30 [3].

On the other hand, the modifiable risk factors of breast cancer include the intake of chosen drugs, physical activity, body mass index, alcohol intake, cigarette smoking, exposure to artificial light, intake of highly processed foods, exposure to chemicals, and insufficient vitamin supplementation [3]. All these factors are crucial to searching for new therapeutic methods for breast cancer. Numerous studies indicate how one’s diet and an imbalanced micronutrient status might significantly affect the carcinogenesis of various cancers, including breast cancer [4,5,6]. A large number of current studies on this matter focus on specific vitamins—such as vitamin C or E [7]; however, most studies are currently focused on vitamin D [8,9,10,11] as its deficiency is very common in patients with breast cancer. Also, a new and currently very promising direction of research on breast cancer therapy methods includes phytochemicals that can induce epigenetic changes in cells [12]. Not only are they important in drug development, but also some of them may even act as natural inhibitors of pathological cell proliferation and support anticancer therapy for breast cancer. Micronutrients, including vitamins and minerals, play crucial roles in various physiological processes and are essential for maintaining one’s overall health. Numerous studies investigated the potential influence of micronutrient status on breast cancer progression. Most of the research emphasizes the importance of further studies and experiments, as an imbalanced micronutrient status may be highly associated with breast cancer progression. Micronutrients may also play an important role in carcinogenesis as well as in the progression of the tumor. Compounds such as vitamin B, vitamin D, vitamin E, or β-carotene interfere in processes like proliferation, immunological response, metastasis, or angiogenesis and modulate the onset of the tumor. Reports indicate that the proper supplementation of micronutrients may be crucial for both the prevention of and therapy for breast cancer [5,13]. In this narrative review, we aimed to summarize the current knowledge regarding the micronutrient status in patients with breast cancer as well as indicate the possible improvements of micronutrient supplementation in order to prevent the progression of carcinogenesis.

## 2. Micronutrients: Vitamins

### 2.1. Vitamin A

Vitamin A, a fat-soluble compound, encompasses retinol and derivatives like retinal, retinoic acid (RA), and carotenoids, including α- and β-carotene, β-cryptoxanthin, lycopene, lutein, zeaxanthin, and canthaxanthin. Three carotenoids, namely, α- and β-carotene and β-cryptoxanthin, can be converted into retinol in the body [14]. The recommended daily allowance for vitamin A among adults is 700 to 900 μg. This amount can be provided by a diet, mainly by red and orange vegetables and fruits, as well as green vegetables, milk and dairy products, eggs, and fish [15,16]. Deficiency of vitamin A might lead to various symptoms and conditions, including nyctalopia, xerophthalmia, or keratomalacia; vitamin A deficiency might be detected using blood tests.

Different forms of vitamin A, including all-trans retinoic acid (ATRA), are approved by the Food and Drug Administration for treating aggressive blood cancer [17]. ATRA is the major biologically active form of vitamin A and may selectively regulate the expression and activity of matrix metalloproteinases (MMPs), such as MMP-2 and MMP-9, in cancer progression and metastasis [18]. A key mechanism used by cancer cells to suppress the immunological response is the sequestration of dendritic cells (DCs) to inhibit their mobility [19]. To counterattack immunosuppression, enhancing DC mobility is a promising strategy [16]. MMPs, specifically MMP-2 and MMP-9, emerge as key players in DC movement through basement membranes and the extracellular matrix. ATRA upregulates MMP-2 and MMP-9 expression and activity, improving DC immune responses [20]. Conversely, different forms of vitamin A exhibit a dual role in regulating MMPs.

In a 4T1 murine breast tumor model, ATRA and alpha-galactosylceramide reduced lung metastatic foci by 70%, providing new evidence of the downregulation of MMP production as a possible mechanism [21]. In neuroblastoma cells, β-carotene suppresses MMP-2 activity and downregulates hypoxia-inducible factor-1α (HIF-1α), a key player in MMP upregulation [22]. Similar inhibitory effects are observed in gastric and colorectal cancer cells, showcasing the potential of vitamin A in mitigating invasive properties [23].

β-Carotene, a prominent carotenoid, emerges as a guardian against breast cancer. Β-carotene’s high serum levels are associated with a 17% reduction in breast cancer risk [24].

Dietary β-carotene intake is significantly associated with improved breast cancer survival, with a 30% reduction in the odds of death according to a meta-analysis conducted by He et al. (2018) [25]. A large prospective study demonstrated that high levels of plasma carotenoids are associated with a 28% lower risk of breast cancer in a 20-year follow-up [26].

Jain et al. (1994) reported a significant association between pre-diagnosis dietary β-carotene intake and prognosis in patients with breast cancer with estrogen receptor (ER+) or progesterone receptor (PR+) status, but not in those with ER- or PR-negative status [27]. RA was found to inhibit the growth of ER-positive breast cancer cells by blocking the cell cycle [25].

ER-negative breast cancer cells express lower levels of retinoic acid receptors (RARs)-β compared to their ER-positive counterparts [28]. This suggests a nuanced relationship between β-carotene, RA, and breast cancer prognosis, with differential effects based on one’s hormone receptor status.

Studies on vitamin A and breast cancer survival report inconsistent results, with some indicating a reduced mortality risk with β-carotene intake. Plasma retinol and vitamin A show no significant relationship with breast cancer [29]. A meta-analysis by He et al. shows that the pre-diagnosis dietary intake of various carotenoids and retinol reveals no significant link to total mortality from breast cancer [25]. Retinoid receptors including RARs and retinoid X receptors (RXRs) exhibit antiproliferative effects, and carotenoids may influence cellular differentiation, proliferation, and immune function.

So far, there is one clinical trial investigating the relationship between the intake of natural RAs and breast cancer onset and progression. In a phase II trial for metastatic breast cancer, the recommended daily dose of 150 mg/m^2^ ATRA for 14 consecutive days did not demonstrate significant activity [30]. Out of the fourteen patients who completed the study, one achieved a partial response, and three had a stable disease. The results indicated a notable interpatient variability but with acceptable toxicity levels.

Overall, vitamin A’s role in breast cancer is complex, with mixed findings. High dietary vitamin A consumption correlates with decreased breast cancer incidence in North American and Asian populations [31]. The dysregulation of the retinoid signaling pathway is linked to tumor development. Cellular retinol binding protein-1 (CRBP-1) downregulation in breast, ovarian, and nasopharyngeal carcinomas is associated with malignancy progression [30]. The exact mechanism in which CRBP-1 downregulation is involved in the progression of various cancers, including breast cancer, was proposed by some researchers who showed that an overexpression of CRBP-1 suppresses cancer stemness and tumorsphere formation as well as generally inhibits tumorigenicity. In breast cancer, for example, where CRBP-1 expression is significantly low, those mechanisms are impaired, leading to further carcinogenesis [32]. Vitamin A derivatives, in synergy with carotenoids, exhibit potential in breast cancer treatment and prevention, warranting further clinical investigation, particularly in combination with chemotherapy.

Carotenoids are common compounds with many physiological roles, such as the initiation form of vitamin A synthesis or antioxidant properties [33]. These biological functions may play an important role in the prevention of and therapy for breast cancer as well. Carotenoids present antioxidant features such as the prevention of peroxidation of adipose tissue or the suppression of the generation of free radicals. Gloria et al. reported the apoptotic effect of carotenoid intake in a group of MDA-MB-231, MDA-MB-235, and MCF-7 cell lines. Their research showed that carotenoids are an important factor in the process of apoptosis and can regulate the expression of twenty-one genes associated with cell death [34,35]. Carotenoids, as the precursor of vitamin A, regulate cell signaling, including signaling in breast cancer cells. RA binds to RXRs and RARs, which are the steroid hormone’s nuclear receptors and one of vitamin A’s properties. RXRs and RARs can modify the transcription of genes that code for numerous enzymes, binding proteins, and structural proteins. By binding to RARα and upregulating the RARβ gene, which, in turn, stimulates other genes involved in the differentiation of cells and apoptosis, retinoids can suppress the spread of breast cancer in vivo [36,37]. Kim et al. presented that retinoids may potentially suppress tumor expansion in patients with ER-positive and ER-negative breast cancer. By suppressing the telomerase and cyclin D levels in ER-positive cancers, retinoids can cause aging and cell cycle blockage. They also promote growth suppression in ER-negative cancers by upregulating the expression of retinoblastoma proteins p21 and p53. Moreover, carotenoids might prevent metastatic lesions’ onset by suppressing RAS/RAF/MEK/ERK1/2 signaling and downregulating the PI3K/Akt/mTOR pathway, which, in turn, prevents cell migration and proliferation [37]. Research has shown that there is a possible correlation between carotenoid consumption and a decreased risk of breast cancer onset [38,39].

### 2.2. Vitamin B

The one-carbon metabolism, a complex network of biochemical pathways involving folate and related B vitamins, plays a crucial role in providing methyl groups for various biological processes [40]. Vitamin B6 and B12 are essential for DNA synthesis, repair, and methylation and can cause uracil and chromosome breaks [41,42]. The dysregulation of this intricate metabolic network is implicated in promoting carcinogenesis [41]. Deficiencies in vitamins from the vitamin B group can lead to inflammation and oxidative stress [43]. The daily supplementation dose for the vitamin B group depends on the type of vitamin. The major dietary sources of this group of vitamins are animal-derived foods, for example, meat, milk, eggs, and fish, as well as dried green and purple lavers [44]. Deficiencies in vitamin B levels might lead to anemia; vitamin B levels can be checked either by blood or urine tests.

The relationship between vitamin B intake and breast cancer risk has primarily been evaluated in several meta-analyses, and studies indicate contradictory results [45,46,47]. A meta-analysis of case–control studies suggested a significant protective effect of folate intake on breast cancer incidence, whereas cohort analyses did not present such effects [45].

Specifically, high serum levels of pyridoxal 5′-phosphate, the active form of vitamin B6, were associated with a 20% reduction in breast cancer risk among postmenopausal women [46]. However, no significant association was found between dietary vitamin B6 intake, serum vitamin B12, and dietary vitamin B12 intake and breast cancer risk [46,47].

Dietary vitamin B2 intake showed a weak reduction in breast cancer risk, and, in stratified analyses, no significant association was found with ER-positive or ER-negative breast cancer risk [45]. Vitamin B2, a cofactor in the one-carbon metabolism, may modulate methyl group bioavailability, influencing DNA stability and integrity [40].

Folate intake might be associated with breast cancer risk, indicating a significant reduction in daily dietary folate intake between 153 and 400 μg compared with <153 μg [46]. However, dietary folate intake exceeding 400 μg did not show a significant reduction in breast cancer risk. The use of folic acid-containing supplements in *BRCA1* mutation carriers significantly decreased the risk of breast cancer by 55% compared to non-users [48].

Studies analyzing the circulating levels of vitamin B12 with respect to cancer outcomes present various results. Elevated cobalamin levels before breast cancer diagnosis have been associated with a higher mortality [49]. However, the relationship between vitamin B12 use before and during chemotherapy and breast cancer outcomes remains unclear.

The dynamic role of folates in carcinogenesis presents a fascinating paradox. Some studies suggest a dual impact on tumor development based on the timing of supplementation, underscoring the preventive potential of folate supplementation when administered before the existence of preneoplastic lesions [50]. Evidence suggests that excess folate during cancer diagnosis can paradoxically contribute to breast tumorigenesis [51,52]. The expression of tumor suppressor genes, such as phosphatase and tensin homolog (PTEN) and adenomatous polyposis coli (APC), is purportedly decreased with the increased methylation of their promoters [51]. High-folate diets are associated with an increase in tumor volume and the number of tumors in MMTV-PyMT mice [53]. Conversely, a deficiency in folates exhibits a significant inhibitory effect on breast cancer.

The transition from preclinical models to human studies introduces a layer of complexity. While one study reports an inverse association between folate consumption and mortality, another fails to establish any significant correlation [54,55]. The intricate balance between folate supplementation and its potential repercussions on gene regulation and tumor dynamics underscores the need for continued research to decipher this intriguing paradox.

### 2.3. Vitamin C

Vitamin C, also known as L-ascorbic acid, functions as a robust antioxidant, neutralizing naturally produced free radicals and those induced by environmental factors [56]. Free radicals contribute to oxidative stress, which is particularly harmful to cellular components [57]. DNA damage stands as a pivotal element in the progression of cancer, as interactions between free radicals and DNA can induce mutations, fostering uncontrolled cell growth [58]. The dietary optimum intake for vitamin C is 200 mg per day. The best dietary sources for vitamin C are tomatoes, potatoes, and citrus fruits [59,60]. Measures used to detect vitamin C deficiency primarily include simple blood tests.

In vitro studies employing pharmacological doses of vitamin C at high concentrations (1 mM) presented cytotoxic effects on tumor cells and impeded tumor growth in vivo without meaningful toxicities to normal cells [61,62]. Experiments conducted in a cell culture showed that elevated plasma concentrations of millimolar vitamin C possess the capability to induce cancer cell death [63,64]. This effect is ascribed to the pro-oxidative activity of ascorbate, culminating in the generation of hydrogen peroxide (H_2_O_2_) and hydroxyl radicals (OH•) [58].

The combination of auranofin and vitamin C has been found to exert synergistic and H_2_O_2_-mediated cytotoxicity toward MDA-MB-231 cells and other breast cancer cell lines [65]. In vivo confirmation of the anticancer potential of auranofin/vitamin C combinations was achieved using MDA-MB-231 xenografts in mice, without notable side effects. The MDA-MB-231 cell line is commonly used to model late-stage breast cancer; it is also useful in terms of identifying genes and pathways responsible for metastasis in various cancers.

Vitamin C demonstrates proficiency in quenching free radicals and reducing oxidative DNA damage, employing both oxidant and epigenetic mechanisms [57,58]. This underscores its multifaceted role in alleviating cellular stress and its potential contributions to the demise of cancer cells.

Several studies elucidated the potential of ascorbic acid to elicit apoptosis in breast cancer cell lines [62,66]. In a study conducted by Hong et al., the capacity of ascorbate to induce apoptosis in breast cancer cells was demonstrated. This intricate process is reliant on caspase activation while also exhibiting self-sufficiency [66]. Within the complex cascade of apoptosis, the mitochondrion responds to pro-apoptotic signals by releasing the apoptosis-inducing factor [66]. The apoptosis-inducing factor initiates its translocation to the cell nucleus, where it instigates chromatin condensation and DNA fragmentation. Notably, vitamin C levels have been correlated with the upregulation of TRAIL, a known apoptosis inducer [67].

Vitamin C showcases anti-inflammatory functions by modulating the cytokine levels, as evidenced by decreased pro-inflammatory cytokine levels (interleukin-6, interleukin-1) in animal models of melanoma and breast cancer following vitamin C administration [68].

However, the precise contribution of vitamin C’s oxidant scavenging function versus its gene-regulatory cofactor’s functions to cytokine modulation remains unclear. Notably, patients with higher inflammation levels appear to have an increased requirement for vitamin C [69].

Vitamin C plays a pivotal role as a cofactor for three hydroxylase enzymes crucial in stabilizing the tertiary structure of collagen [70]. The significance of vitamin C in impeding the progression of cancer metastasis is closely tied to its involvement in collagen synthesis, a process which undergoes a decline in the absence of sufficient vitamin C [71].

Notably, studies have presented similarities in the stromal alterations surrounding a tumor to those observed in scurvy [68]. Consequently, a dense stromal consistency acts as a physical barrier, restricting the spread of neoplastic cells by encapsulating them within a dense fibrous tissue. Studies investigated the impact of low-dose oral vitamin C supplementation on the collagen encapsulation of tumors in Gulo knockout mice with melanoma and breast tumors [68]. The findings demonstrated an increase in collagen encapsulation, suggesting a potential role of vitamin C in modulating the tumor microenvironment and influencing the physical barriers which constrain the spread of cancer cells.

In recent studies, derivatives of ascorbic acid have been presented as promising contenders in the battle against breast adenocarcinoma. Macan et al. (2019) showcased the potent cytostatic activity of 6-(1,2,3-triazolyl)-2,3-dibenzyl-L-ascorbic acid analogs against breast adenocarcinoma cell lines, regulating the hypoxia-inducible factor (HIF-1α) signaling pathway with minimal toxicity to normal fibroblasts [72]. Another study by Harej et al. (2019) introduced new 4-substituted 1,2,3-triazole L-ascorbic acid analogs, exhibiting highly selective antiproliferative activity against breast adenocarcinoma [73]. Moreover, investigations into parenteral ascorbate have hinted at its synergistic potential with chemotherapy or radiotherapy, enhancing the quality of life of patients with cancer by safeguarding normal tissues [74].

The structural similarity between vitamin C and glucose facilitates the former’s transport through glucose transporter 1 (GLUT1) [75]. Breast cancers with higher grades exhibit an upregulation of GLUT1 expression, allowing the entry of dehydroascorbic acid (DHA) [76]. Tumor cells with elevated GLUT1 levels internalize DHA, inducing intracellular oxidative stress and oxidative damage through the reduction of DHA back into ascorbate.

Ascorbate, a key player in fortifying the immune system, inhibits the synthesis of interleukin 18 (IL-18), a stimulator of interferon-gamma synthesis in immune cells [74]. IL-18 also promotes the expression of transferrin, a positive regulator of cell growth and proliferation in breast cancer cells [77]. Therefore, ascorbate’s potential efficacy against cancer lies in its ability to diminish IL-18 expression, influencing the immune system’s capacity to counteract diverse tumor cells, including breast cancer cells [78,79].

The exploration of high-dose vitamin C as a potential treatment for breast cancer finds its roots in in vitro studies, particularly involving breast carcinoma cells like MCF-7, which exhibit sensitivity to elevated vitamin C doses [80]. Vitamin C deficiency is associated with increased tumor growth, while supplementation reduces cell proliferation [80,81].

Despite varying vitamin C statuses in patients with breast cancer based on their cancer stage, a meta-analysis of 17.000 patients indicates that the post-diagnosis utilization of antioxidant vitamin supplements, including vitamin C, does not exhibit a significant association with the overall mortality [82]. However, the intake of vitamin C supplements after diagnosis shows a statistically significant correlation with a reduced risk of total mortality. A meta-analysis of case–control studies suggests a protective impact associated with elevated doses of vitamin C against the risk of breast cancer, although cohort studies have not demonstrated a significant preventive influence [83,84].

In women with obesity, an association between vitamin C intake and breast cancer has been observed [74]. Elevated estrogen levels in postmenopausal women with obesity are linked to the chronic inflammatory state associated with obesity, resulting from the conversion of testosterone to estrogen in adipose tissue through aromatase [85].

Studies exploring the potential synergistic effects of combining high-dose vitamin C with other agents for breast cancer treatment demonstrate a dose-dependent inhibition effect on breast cancer cell proliferation [86,87,88,89]. The consistent outcomes strongly support the idea that high-dose vitamin C enhances the therapeutic effectiveness of different chemotherapy agents [74]. Additionally, the observed extra impact on chemotherapy-resistant breast cancer cells suggests that high-dose vitamin C independently inhibits breast cancer proliferation. Given the common development of resistance to conventional anticancer therapies in advanced breast cancers, high-dose vitamin C presents a promising strategy to overcome such a resistance [63].

### 2.4. Vitamin D

Vitamin D deficiency, defined as a serum 25-hydroxy vitamin D level below 20 ng/mL or 50 nmol/L, is prevalent in all patients with breast cancer, with a particularly higher incidence in those with triple-negative breast cancer (TNBC), the most aggressive form [90,91]. A daily vitamin D supplementation dose for the adult general population is 2000 IU (50 µg). Good dietary sources for vitamin D are fish (not only fatty fish), egg yolk, and offal such as liver, as well as fortified milk [92,93]. The most approximate way to measure the vitamin D levels in the organism is to specifically perform the 25-hydroxy vitamin D test.

Calcitriol (1,25-dihydroxy vitamin D; 1,25(OH)_2_D), the active form of vitamin D, exhibits antiproliferative activities and promotes the differentiation of hematopoietic cells through the vitamin D receptor, a nuclear receptor transcription factor [94]. Malignant breast cancer cells have shown the downregulation of vitamin D receptor, limiting their responsiveness to the antitumor activity of vitamin D [94,95]. Vitamin D gained interest for its potential as a breast cancer preventative agent, with an association established between plasma concentrations of vitamin D and breast cancer carcinogenesis, along with better outcomes for diagnosed individuals [96]. There is substantial evidence supporting the protective role of vitamin D against breast cancer development [97,98,99], mostly in preclinical studies.

Contrary to some expectations, large, randomized trials, such as the Women’s Health Initiative and the Vitamin D and Omega-3 Trial (VITAL), did not demonstrate a significant reduction in breast cancer incidence with vitamin D supplementation [100,101,102]. Similarly, a meta-analysis of 70 observational studies found no significant association between a 400 IU/day increment in vitamin D intake and breast cancer risk overall, though there may be a potential decrease in the risk for Asian and premenopausal women [9]. The relationship between vitamin D and breast cancer may be influenced by genetic factors. Some studies have explored the interactions between specific genetic polymorphisms and vitamin D with respect to breast cancer risk and outcomes in Asian women [103,104].

Studies have identified vitamin D receptors and CYP27B1 and CYP24A1 enzymes responsible for synthesizing and metabolizing calcitriol in both normal human breast tissue and breast cancers [94]. Vitamin D can modulate the influence of endogenous estrogen, a known breast cancer promoter, by reducing aromatase expression and estrogen production in breast adipose tissue and downregulating ERα expression [105].

Experimental studies have explored the antiproliferative effects of vitamin D on cancer cells, including breast cancer cells, indicating a potential role in regulating cell growth and promoting apoptosis [106]. The exact mechanism linking vitamin D to breast cancer risk remains unclear, with some studies suggesting an inverse relationship between vitamin D status and breast cancer risk.

While vitamin D shows promise in preclinical studies, the complex interplay of genetic factors, hormonal influences, and the specific microenvironment of breast tissue adds layers of complexity to its role in breast cancer prevention and treatment. Further research is needed to delineate the intricate mechanisms and identify specific subgroups that may benefit from vitamin D interventions.

### 2.5. Vitamin E

Vitamin E is an antioxidant, and it has been investigated for its potential role in reducing oxidative stress and inflammation, which are factors associated with cancer progression. However, the evidence is not conclusive, and further research is required [107,108]. A daily allowance for vitamin E is 15 mg per day among adults. The best food sources are fortified ready-to-eat cereals, nuts, seeds, greens, and vegetable oils [109]. Any deficiencies in the levels of vitamin E might be detected using simple blood tests. Both tocotrienol studies showed antitumor properties due to the inhibition of growth, invasiveness, and migration. Furthermore, tocotrienol may be an important feature in combined chemotherapy against breast cancer. Research has revealed that combined etoposide and γ-tocotrienol present antitumorigenic properties and successfully reduce breast cancer cell proliferation [110,111]. Moreover, a combined breast cancer therapy of etoposide and γ-tocotrienol may not be the only promising therapeutic path. Trujillo et al. pointed out the positive effects of a combined therapy of tocotrienols with celecoxib, gefitinib, or erlotinib [112]. Kline et al. presented the possible mechanisms of the antitumorigenic properties of different forms of vitamin E. By both the overexpression of the major cell cycle regulatory protein p21 and the stimulation of ERK1 and MEK1 kinases, vitamin E succinate causes G0/G1 cell cycle block and inhibits the process of proliferation [113]. Also interesting is the fact that γ-tocotrienol may inhibit the onset of metastasis. γ-Tocotrienol suppresses the Rac1/WAVE2 signaling pathway and leads to the prevention of tumor expansion [114]. Another argument for the crucial role of vitamin E in breast cancer development that should be taken into consideration is the positive correlation between severe α-tocopherol deficiency and increased breast cancer risk. Hu et al. observed a significant association between plasma α-tocopherol and breast cancer in a subgroup with a median lowest level of 5.74–9.16 μmol/L [29].

### 2.6. Vitamin K

Vitamin K is a compound crucial for physiological actions such as gamma-carboxylation of different proteins including proteins essential for coagulation processes. Moreover, vitamin K may also be involved in the process of carcinogenesis [115,116]. The current daily recommended dietary allowance for vitamin K is 1 µg/kg, while the best food sources are leafy green vegetables and vegetable oils (soybean, cottonseed, canola, and olive) [117]. In order to assess any deficiencies regarding vitamin K levels, blood tests are performed in which coagulation screening tests enable the clinicians to indicate early or mild vitamin K deficiency. Yamada et al. reported the antitumorigenic effect of vitamin K supplementation on breast cancer in a rodent model. By affecting cellular nucleophiles, including protein cysteine residues, and thanks to the generation of reactive oxygen species (ROS), vitamin K3 (VK3) inhibits breast cancer expansion no matter the molecular subtypes established by receptor overexpression. The ROS generated by VK3 may induce the stimulation of extracellular signal-regulated kinases (ERKs), which promote the emergence of death-promoting factors. Furthermore, by separating PARP or caspase-3, VK3 initiates the apoptotic cascade and interrupts the cell cycle [118,119]. Research has shown that vitamin K2 (VK2) presents promising features in TNBC therapy. Kiely et al. reported the suppression of adhesion up to 70% of MDA-MB-231 and MDA-MB-453 cells due to VK2 intake. Their study suggests that a half-maximal inhibitory concentration (IC50.) for the MDA-MB-231 cell line is approximately 124.37 μmol/L [120]. In another study, VK3 also presented a cytotoxic effect through the disintegration of nuclear DNA on the MCF-7 human breast cancer cell line. Akiyoshi et al. reported the influence of VK3 intake on the activity of caspase-7 and caspase-9, on which the process of apoptosis is dependent [121]. Studies describing the involvement of the aforementioned vitamins in the onset and progression of breast cancer are presented in Table 1.

## 3. Micronutrients: Minerals

### 3.1. Copper

Copper (Cu) is a trace element essential for the proper functioning of the body [122]. The recommended dietary intake of copper is 900 µg/day, while the best food sources are shellfish, whole grains, beans, nuts, potatoes, and organ meats (kidneys, liver) [123]. It is involved in the cellular metabolism as a catalytic cofactor in the regulation of energy production, oxygen transport, iron storage, signal transduction, and other intracellular processes. Even a small increase in Cu concentration in the body can cause severe toxicity, oxidative stress, initiation, and promotion of carcinogenesis [124,125]. The 2022 case–control study by Barartabar et al. showed that the Cu levels (*p* = 0.0001) in tumor tissue were significantly higher than in tumor margin tissue in 40 women with a histologically confirmed diagnosis of invasive ductal carcinoma [122]. Ceruloplasmin tests might be helpful in diagnosing Cu deficiencies.

A meta-analysis conducted in 2020 by Feng et al. showed that the Cu levels in the serum of patients with breast cancer were significantly higher than in healthy controls and patients with benign breast disease, which could be responsible for the rapid growth of tumor cells [126].

There are many theories on how increased Cu levels contribute to carcinogenesis. One of them is the Cu-dependent cell death mechanism, cuproptosis [127]. This process occurs mainly in cells with increased levels of lipoylated enzymes of the tricarboxylic acid cycle, where metabolic processes are intense. In these cells, Cu and fatty acylating components are combined, fatty acylated proteins become aggregated, intracellular toxic oxidative stress is initiated, and cell death occurs as a result [127]. A 2022 study by Huang et al. demonstrated the association of cuproptosis with breast cancer progression based on an analysis of the data and expression patterns of cuproptosis-related genes (CRGs) in breast cancer tissue and normal breast tissue using the University of Alabama at Birmingham Cancer Data Analysis Portal (UALCAN) [124]. It was shown that the expression of the following CRGs—CDKN2A, PDHA1, and lipoyltransferase 1 (LIPT1)—was associated with breast cancer incidence [124]. It was also shown that PDHA1 overexpression was significantly associated with a negative prognosis in patients with breast cancer due to low overall survival (OS) and low recurrence-free survival (RFS) rates [124]. Shi et al. (2023), using bioinformatics analysis, identified 11 CRGs associated with the development of TNBC: NFE2L2, NLRP3, ATP7B, ATP7A, SLC31A1, LIAS, LIPT1, DLAT, PDHB, GLS, and DLST [116]. Based on these, an 11-gene risk model effective in predicting 5-, 10-, and 15-year survival in patients with TNBC was also developed [125].

The role of CRG LIPT1 in carcinogenesis was established in a 2022 study by Liu et al. [128]. This gene encodes LIPT1, a lipoic acid-specific sequence enzyme responsible for maintaining the oxidative and reductive metabolism of glutamine [129]. The overexpression of LIPT-1 was associated with a favorable prognosis in patients with breast cancer, and it was also correlated with immune cell infiltration (B lymphocytes, fibroblasts, and CD8+ T lymphocytes) [128]. A role for LIPT-1 as a novel prognostic and immunologic biomarker in breast cancer has been proposed [128]. An analysis of the Molecular Taxonomy of Breast Cancer International Consortium (METABRIC) 2000 dataset showed that patients with breast cancer with a high expression of the CRG SLC31A1 had a worse prognosis than those with a low expression of this CRG [130]. It was also shown that SLC31A1 was correlated with the histological subtype of breast cancer, as patients with aggressive TNBC and human epidermal growth factor-2 (HER2+) cancer subtypes had higher SLC31A1 expression than patients with luminal A and B cancer subtypes [130].

CRGs are involved in modifying the tumor microenvironment and immune response in breast cancer [131]. Patients with breast cancer with a high cuproptosis-related gene score (CRG_score) have an unfavorable prognosis, while patients with a low CRG_score are sensitive to immunotherapy [131]. The CRG_score can be used to evaluate the expression pattern of CRGs and the corresponding features of immune cell infiltration in breast cancer to assess the immunophenotype of the tumor, and it can also be used as an independent prognostic biomarker to indicate the efficacy of possible immunotherapy [131,132]. Copper chelation is one of the proposed therapeutic strategies for breast cancer. A 2017 phase II clinical trial by Chan et al. demonstrated the efficacy of the oral Cu chelator, tetrathiomolybdate, in 75 patients with breast cancer at a high risk of recurrence [133]. The OS was 84% with a median follow-up of 6.3 years in these patients [133].

### 3.2. Chromium

Chromium (Cr) is a trace element, naturally found in the Earth’s crust, soil, and rocks. Cr (III) compounds are harmless to humans and are used as dietary supplements. Cr (VI) compounds are classified according to the International Agency for Research on Cancer and the United States Environmental Protection Agency (EPA) as group 1 and group A carcinogens, and exposure to them in humans is widespread due to the chemical industry and environmental pollution [134,135]. The estimated daily dietary intake for chromium is 50–200 µg, while its best dietary sources are shellfish, broccoli, meat, and nuts [136]. If any suspicions regarding possible chromium deficiencies arise, usually Cr blood tests are conducted. However, in cases where it is possible that a patient might be exposed to toxic hexavalent Cr or if there are suspicions of poisoning, urine tests are usually performed in such circumstances.

**Figure 1:**
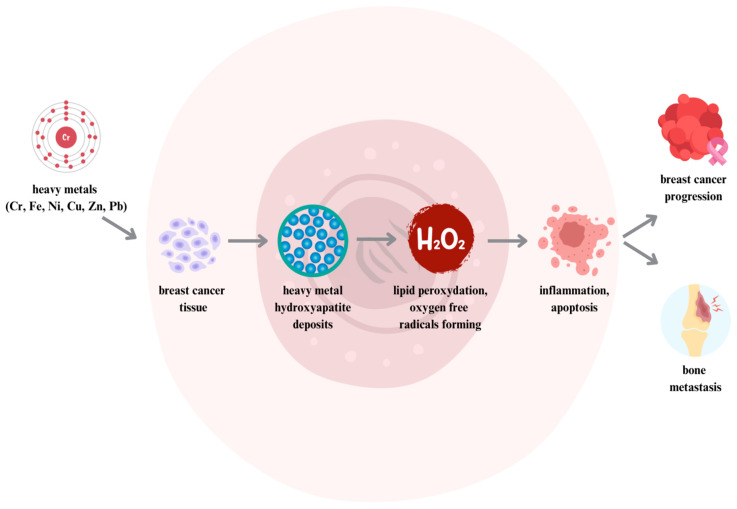
Pathological biomineralization in breast cancer tissue.

A 2022 meta-analysis by Batyrova et al. indicated that the Cr levels in hair samples from female patients with breast cancer were significantly elevated in comparison to samples from healthy women [137]. One of the proposed theories of how Cr contributes to breast cancer is that the element may affect the levels of lipids and other small-molecule metabolites in the blood that may be involved in the development of breast cancer [138]. Elevated Cr levels can lead to lipid peroxidation and the increased formation of free radicals and reactive oxygen species and can also activate estrogen receptors, resulting in the promotion of breast cancer cell proliferation [135,137,138,139]. A 2016 study by Romanjuk et al. showed the involvement of a pathological tissue biomineralization process in breast cancer progression [139]. Chromium and other heavy metals entering tumor tissues replace calcium in hydroxyapatite particles, initiating local inflammation in the area of the foreign body and increasing cell division, the synthesis of MMPs, and the formation of bone metastases [139]. Biomineralization and intense inflammatory infiltration in ductal breast cancer are unfavorable histological markers [137]. The process of pathological biomineralization and its consequences in breast cancer are shown in Figure 1.

Elevated Cr levels can cause DNA damage and Cr-stimulated structural genetic changes (including inter-strand cross-links, DNA protein cross-links, strand breaks, and Cr-DNA adducts) [135,137]. A 2023 study by Sawicka et al. aimed to evaluate the effects of Cr (VI) interaction with 17β-estradiol and its metabolites on cytotoxicity and DNA damage in vitro in breast cancer cell lines [135]. It was found that the two agents separately induced cell apoptosis, while Cr (VI), in combination with 17β-estradiol’s metabolite, 2-methoxyestradiol, caused increased apoptosis and DNA damage larger than both agents evaluated separately [135].

### 3.3. Cobalt

Cobalt (Co) is a trace element widely used in the chemical industry. It is also a component of orthopedic prosthetic implants that may cause Co-related toxicity [140]. The dietary intake of cobalt varies between 5 and 50 μg/day, while its food sources are fish, nuts, broccoli, and spinach [141]. Co salts are recognized carcinogenic agents of grade 2A and 2B in the International Agency for Research on Cancer classification [142]. The possible mechanisms responsible for Co carcinogenicity include the induction of oxidative stress and the generation of reactive oxygen species, damage to mitochondrial DNA, induction of cell apoptosis, enzyme inhibition, and the upregulation of the HIF-1α. Co has also been shown to activate estrogen receptors and stimulate breast cancer cell proliferation [137].

Hypoxia is one of the most common disorders in the tumor microenvironment, promoting further cancer growth [143]. HIF-1α is a transcription factor activated under hypoxia [143]. This factor can promote tumor growth by inducing erythropoietin synthesis, angiogenesis, and glucose uptake [140]. Cobalt chloride (CoCl_2_) is widely used in research as a chemical inducer of hypoxia. A 2016 study by Chu et al. aimed to determine the effect of CoCl_2_-induced hypoxia on carbonic anhydrase (CA IX) protein and mRNA expression as well as on the invasive potential in breast cancer cell lines [144]. CoCl_2_ has been shown to mimic the hypoxic state by increasing the expression of mRNA and the CA IX protein associated with cell migration and cell invasion in vitro, while the CA IX protein itself, due to these properties, can be used as a clinical biomarker to predict tumor progression and metastasis [144]. A 2019 study by Rana et al. additionally showed that CoCl_2_ in breast cancer cells stimulates the expression of the gene for a vascular endothelial growth factor (VEGF) responsible for angiogenesis, the proapoptotic gene BAX, and induces p53, also responsible for cell apoptosis [145].

Polyploid giant cancer cells (PGCCs) are a subpopulation of cancer stem cells present in breast tumors; their formation is induced by various factors, including CoCl_2_ [146,147]. The number of PGCCs is higher in malignant and metastasis-forming tumors than in benign tumors, and their high levels are associated with a poor prognosis for the patient [148]. A 2019 study by Liu et al. showed that the overexpression of cell cycle-related proteins (p38MAPK, ERK, JNK, and CDC25C) can promote PGCC formation to facilitate invasion and metastasis in breast cancer, worsening patient prognosis [146].

### 3.4. Boron

Boron is a trace element, naturally found in plants and drinking water. The daily intake of boron varies between 0.3 and 41 mg per day, and it is obtained from a diet rich in fruits, vegetables, nuts, and legumes [149]. In industry, it is used in the form of boronic acid, which is toxic to humans [150]. Boron compounds are used in cancer treatment: bortezomib, containing boronic acid, is the standard treatment for multiple myeloma, mantle cell lymphoma, and non-Hodgkin lymphomas [151].

A study from 2023 by Mohammed et al. aimed to determine the potential effects of boron derivatives, sodium pentaborate pentahydrate, and sodium perborate tetrahydrate on breast cancer cell lines, PD-1/PD-L1 expression, and activated T cells [152]. Their anticarcinogenic effects have been demonstrated in both TNBC cells and ER+ cancer cells [152]. Both compounds induced apoptosis in breast cancer cells through the downregulation of the monopolar spindle-one-binder 1 (MOB1) protein, as well as antiproliferative effects by inducing a decrease in the expression of the CYR61, CTGF, and BIRC-5 genes [152]. Sodium pentaborate pentahydrate and sodium perborate tetrahydrate can inhibit breast cancer progression but also have the potential to cause side effects by stimulating PD-1/PD-L1 expression and inhibiting the release of cytolytic cytokines [152].

Boron compounds—sodium borocaptate and boronophenylalanine (BPA)—are used in boron neutron capture therapy (BNCT) for the treatment of breast cancer [152,153,154]. The therapy involves the targeted delivery of boron compounds to tumor tissues, with subsequent irradiation with epithermal neutrons [153,154]. BNCT has shown potentially better effects in cancer therapy than standard X-rays due to the formation of irreversible DNA double-strand breaks in tumor cells through an oxygen-independent process [153]. This therapy is dedicated especially to patients with inoperable disease at an early stage, post-radiotherapy patients with locoregional recurrent disease, and metastatic disease [153]. The use of BPA as a boron carrier is most effective in breast cancer with the overexpression of the L-amino acid transporter because BPA enters tumor cells through these exchangers [153]. However, the use of sodium borocaptate and BPA is limited due to significant variability in tumor cell uptake and a low efficiency in cells with low L-amino acid transporter expression [153,155]. A study from 2022 by Utomo et al. evaluated the in vitro cytotoxicity of PGB-0-ol, a water-soluble, novel potential boron carrier in BNCT, against breast cancer cell lines [154]. PGB-0-ol was created by synthesizing PGB-0-F (fructose) and PGB-0-Sor (sorbitol) complexes containing pentagamaboronon-0 (PGB-0), a water-insoluble boron-containing analog of curcumin [154]. PGB-0 has demonstrated an affinity for ER cells, HER2 cells, and TNBC cells [154]. The study demonstrated the efficacy of PGB-0-ol as a B-carrier in BNCT, while the increased cellular cytotoxicity of PGB-0-ol compared to PGB was likely due to the compound’s increased solubility and enhanced uptake by tumor cells [154].

The boron derivative hexagonal boron nitride (h-BN) has been used in the synthesis of water-soluble materials that can serve as carriers for anticancer drugs [156]. Recent reports have indicated that peroxidase mimetic nanozymes that convert H_2_O_2_ into reactive oxygen species that destroy cancer cells could be used in cancer treatment [157]. In a 2021 study by Zeng et al., a nanozyme based on a biodegradable boron oxynitride (BON) structure was developed [157]. BON was shown to reduce breast cancer cell viability by 82% by inducing apoptosis, while BON’s efficacy was confirmed using a mouse model by achieving a tumor growth inhibition of 97% [157]. Catalyzing the production of reactive hydroxyl radicals from H_2_O_2_ in the lysosomes of breast cancer cells, leading to cell apoptosis, has been postulated as a possible BON mechanism of action [157].

In addition to the inhibition of breast cancer progression and the antitumor effects of boron and its compounds, recent studies indicate its potential use in the adjunctive treatment of patients with breast cancer [158]. Radiation therapy is widely used in the treatment of breast cancer, and the most common complication is radiation dermatitis, which occurs in most patients undergoing radiotherapy [159]. A phase III clinical trial in 2022 by Sahin et al. demonstrated the efficacy of a boron-based gel in preventing acute dermatitis, erythema, dry desquamation, and moist desquamation in patients undergoing radiation therapy for breast cancer [158]. The incidence of radiation dermatitis in the patients in the study group was only 9.9% compared to the 98.7% in the control group, making treatment in these patients more comfortable.

### 3.5. Selenium

Selenium (Se) is a trace element essential for humans. It is a component of selenoproteins, which have been associated with antioxidant and DNA-stabilizing properties and, thus, anticancer effects [160]. It has also been suggested that selenium compounds in combination with anticancer drugs can reduce the proliferative activity of cancer cells, improving a patient’s prognosis [161]. The Recommended Dietary Allowance for selenium is 55 µg (0.7 µmol)/day, and it is mostly obtained from seafood, organic meats, and nuts [162]. Regarding the assessment of a possible Se deficiency, this can be diagnosed by simple blood tests; however, these tests primarily indicate the most recent Se intake. In order to assess the long-term Se status, it is preferable to check hair or nail samples.

Data regarding the correlation between Se levels and breast cancer risk as well as patient survival are inconsistent. A meta-analysis of 2021 by Zhu showed a negative correlation between Se levels (serum, nail, plasma) and breast cancer risk (*p* < 0.001, *p* = 0.021 and *p* = 0.014) and no correlation between hair Se levels and breast cancer risk (*p* = 0.092) [161]. A 2021 Polish cohort study by Szwiec et al. of 538 patients with breast cancer showed that low serum Se levels at the moment of diagnosis were associated with an increased risk of death over the next 10 years [163]. The lower mortality rate in women with high serum Se levels at the moment of diagnosis was also confirmed by the Swedish cohort study of 2020 by Sandsveden et al. in a group of 1066 women with breast cancer [164]. However, an American study from 2020 by Guo et al., using Women’s Health Initiative data on 9487 breast cancer cases, found no association between the total Se levels, dietary Se content, or Se supplementation and breast cancer incidence in postmenopausal women [165]. The reason for these different results is the baseline Se level in the population—in the study by Szwiec et al., the average Se level in Polish patients was 86.2 μg/L (with the lowest level being less than 76.8 μg/L), while, in the United States, the average Se level in postmenopausal women was 134.7 μg/L [163]. The Se levels in the Swedish population are comparably low to those in Poland, and the reason for the differences between the populations of different countries is the initial content of the element in the soil, diet, and supplementation in the population [163].

There are many theories on how Se exhibits its anticancer effects. In addition to selenoprotein activity, Se can antagonize the growth of cGMP and inhibit DNA, RNA, and protein synthesis in cancer cells [161]. A 2017 study by Jablonska et al. conducted on breast cancer tissue sections from 42 women found that the dysregulation of Se homeostasis and the accumulation of cadmium in tissues are associated with the development of breast cancer and that breast cancer metastases are most often localized in tissues with a low Se content [166]. A 2021 study by Woo et al. showed antitumor effects of Se in HER2+ breast cancer cells based on the cell lines [167]. Se in trastuzumab-resistant cells downregulated Akt, while the combination of trastuzumab and selenium inhibited beclin-1-related autophagy in trastuzumab-resistant breast cancer cells [167].

Considering the proven anticancer effects of Se, its widespread deficiency worldwide, and the possible carcinogenesis when Se is deficient in the body, it is necessary to supplement this micronutrient and ensure that women achieve the recommended daily intake levels from early in life [161,168]. The daily intake of Se, as recommended by The European Society for Clinical Nutrition and Metabolism (ESPEN), should range from 20 μg/day to 90 μg/day [169]. Because of the risk of the side effects associated with high levels of Se in the body, like nausea, diarrhea, an increased risk of carcinogenesis, diabetes, glaucoma, and even death, Se supplementation should be conducted under professional supervision [161,170].

### 3.6. Manganese

Manganese (Mn) is a trace element, a cofactor for many enzymes, and essential for tissue development; it also regulates neuronal function and immunological processes [171]. The dietary daily intake of manganese among adults is 2 mg/day, and it is mostly obtained from whole grains, clams, oysters, mussels, nuts, soybeans and other legumes, rice, leafy vegetables, coffee, tea, and many spices, such as black pepper [172]. A 2015 meta-analysis by Shen et al. showed an association between Mn deficiency and the development of breast cancer [173]. People with breast cancer had lower serum Mn levels than the healthy controls and a similar correlation was also found using hair samples [173]. Disruption of the balance of the oxidant/antioxidant system has been proposed as a possible mechanism for how Mn deficiency may lead to carcinogenesis [173]. Any Mn deficiencies might be detected using simple blood tests.

Mn compounds are also associated with carcinogenic effects. Natural sources of manganese are cereals, whole grains, and nuts, but it can also be found in contaminated drinking water, thus increasing exposure in humans [174]. Human exposure to high concentrations of Mn in the environment may increase the risk of TNBC progression, while manganese superoxide dismutase (MnSOD) is thought to be a factor in tumor cell invasive activity [174]. Traditionally, MnSOD has been associated with antioxidant activity, but it has been shown that the acetylation status of the enzyme is a molecular switch, making acetylated MnSOD (MnSOD-Ac) act as an oncoprotein in advanced stages of breast cancer [175]. It has also been shown that the abnormal hyperacetylation of MnSOD at lysines 68 and 122 may play a role in aging-related cancers, including breast cancer [176].

Mn and its compounds are being tested for their possible use in the treatment of breast cancer. Manganese-12-acetate is a magnetically bistable molecule showing a combination of strong magnetic anisotropy and high-spin properties [177]. The compound has been shown to have the potential for therapeutic applications by inhibiting migration, invasion, and epithelial–mesenchymal transition (EMT) of breast cancer cells through the suppression of the Wnt/β-catenin and PI3K/AKT signaling pathways and by reducing PD-L1 expression [177]. A Mn2+-releasing nanoplatform has also been developed [171]. Its use together with chemotherapy has shown a strong synergistic effect against breast cancer cells in a mouse model, and it is also possible to use the nanoplatforms in the magnetic resonance imaging of tumors [171].

### 3.7. Molybdenum

Molybdenum (Mo) is a trace element, and, in the human body, it acts as a cofactor of molybdoenzymes [178]. The dietary allowance for molybdenum is 45 µg/daily, and it is mostly found in legumes, dairy, potatoes, and beef liver [179]. Mo deficiencies might be evaluated using various tests, including measuring urinary Mo concentrations, urine sulfite concentration (which is increased in the case of Mo deficiency), as well as blood/urine uric acid level (which is decreased in the case of Mo deficiency). This element is widely used in the imaging diagnosis of breast cancer [180,181]. Molybdenum target X-ray is an inexpensive and quick diagnostic method in oncology, but its limitations include low penetrability and difficulty in imaging tumor borders [180]. Combining this method with magnetic resonance imaging increases diagnostic sensitivity and accuracy [175]. Molybdenum target X-ray can also be combined with b-scan ultrasonography to increase the efficiency of breast cancer diagnosis [181].

A recent report indicated the possibility of using Mo compounds in the formation of anticancer drug carriers for breast cancer [182]. Molybdenum disulfide (MoS_2_) and barium titanate (BT) core–shell nanoparticles (MoS_2_@BT CSNPs) can be used as a photothermal agent and drug carrier for the synergistic therapy (combining chemotherapy and hyperthermia) of breast cancer [182]. A nanocomposite named MoS_2_@BT-PDA-FA (MBPF) was synthesized using polydopamine and folic acid [182]. Inside the particle, gemcitabine was placed, giving a nano-drug which showed antitumor efficacy against TNBC cells [182].

### 3.8. Zinc

Zinc (Zn) is an important trace element involved in many different physiological processes. It plays an essential role in preserving homeostasis, including structural, immunological, or catalytic processes and genome stability. Zn is crucial for the biological function of several proteins and enzymes, cell signaling, microtubule formation, and apoptosis as well as for gene transcription and the proliferation of cells [183,184,185]. The approximate recommended daily intake of zinc is 15–25 mg per day, while the best food sources are meat, grains, cereals, and dairy products [186]. Zn deficiencies might be detected by either blood or urine tests, as well as by hair analysis. Research has shown a relationship between Zn level fluctuations and increased breast cancer risk. Firstly, it has been confirmed that Zn affects the function of the immune system by preventing the DNA from creating ROS and decreasing oxidative stress. Due to immunological modulation properties, Zn influences both the development and the progression of cancers [187]. Several studies demonstrated increased levels of Zn in breast cancer cells compared to normal breast tissue. The regulation of intracellular and extracellular Zn levels that is accomplished by tissue-specific zinc transporters might play an important role in breast cancer development. As one of the zinc transporter families, Zrt-Irt-like protein 6 (ZIP6) is essential in modulating the processes of apoptosis and the EMT, which promotes the survival of cells and the progression of cancer. As a result, it has been observed that a high expression of ZIP6 is considered a trustworthy indicator of luminal A subtypes in breast cancer. Moreover, ZIP6 expression is a predictor of tumor stage and grade and has a major impact on the biological activity of breast cancer cells [187,188,189,190]. Furthermore, cancer progression and metastasis can be accelerated by the impairment of MMPs, especially MMP2 and MMP9, which are a subgroup of Zn-dependent MMPs. Several studies reported EMT stimulation and the accelerated expansion of tumors due to increased MMP activity [188,191]. Holanda et al. reported the direct as well as indirect role of MMPs in tumor growth. MMPs stimulate the growth of breast cancer cells and their spread to other locations by impairing the basal membrane and extracellular matrix. MMPs can also stimulate angiogenesis and lead to accelerated neoplasm dissemination by its better nourishment [192]. Research has shown a correlation between Zn imbalance and the progression of breast cancer. Zinc’s modulating potential on oxidative stress may prevent DNA damage, which is one of the cancer development risk factors. Furthermore, the functioning and expression of impaired zinc transporters may result in extended breast cancer cell survival and lead to more aggressive expansion of the tumor [189,193,194,195]. Research has shown that zinc nanoparticles may be considered a potential treatment strategy in patients with breast cancer. Zinc nanoparticles may stimulate the synthesis of cytokines, such as the tumor necrosis factor-α (TNF-α), which plays an important role in cancer development prevention. Studies on animal models showed the inhibition of tumor growth as a result of zinc nanoparticle application [196,197].

### 3.9. Iron

Iron (Fe) is an essential micronutrient for human health; iron helps in a variety of biological functions and is crucial to homeostasis, including proper oxygen transport to the cells, and mitochondrial activities [198,199]. The recommended daily intake of Fe is 8 mg for adult males and 18 mg for adult females, while its best food sources are lean meat, seafood, nuts, beans, vegetables, and fortified grain products [200]. Fe deficiency primarily leads to anemia, which might be detected by blood tests which should include a complete blood count. The role of Fe in breast cancer onset might be different in specific conditions. Research has shown that an elevated level of Fe may result in an increased risk of breast cancer development in a group of postmenopausal women, while Fe deficiency may be a factor contributing to the increased recurrence of breast cancer in premenopausal women. Fe plays an important role in oxidative processes, including catalyzing the Fenton and Haber–Weiss reactions which result in the generation of ROS. Moreover, studies have pointed to elevated levels of Fe after menopause. In conjunction with increased levels of estrogen, Fe can induce redox reactions and lead to the transformation of Fe^3+^ to Fe^2+^. All these reactions may result in oxidative DNA damage and subsequent carcinogenesis [201,202]. Huang pays attention to the correlation between high levels of estrogen and a co-existing Fe deficiency in premenopausal women. Such a situation may lead to increased VEGF production, which plays an important role in angiogenesis and the following tumor expansion and metastasis [202]. Research has shown that significantly exceeding the recommended Fe intake might result in an increased risk of breast cancer onset. Fe overload has been linked to increased oxidative stress and immune system dysregulation, which are important factors contributing to an elevated risk of breast cancer [203,204]. Huang et al. pointed out increased cancer onset, including breast cancer associated with great red meat consumption. The results revealed that the high concentration of Fe in processed and red meat significantly contributes to a more frequent breast cancer occurrence [205].

### 3.10. Calcium

Calcium (Ca) is one of the most important microelements for the human organism, with a wide range of biological activities, including cell signaling, regulation of the electrical activity of the heart, and skeletal modulation. Ca is necessary for the physiological functions of the skeletal, cardiovascular, and endocrine systems, providing the maintenance of homeostasis [206,207,208]. The recommended daily value for calcium is 1300 mg for adults and children older than four years of age, while its best dietary sources are milk and other dairy products [209]. In order to assess Ca deficiency, serum calcium should be evaluated by conducting blood tests. Research has shown that Ca plays a crucial role in the process of apoptosis. Increased levels of calcium caused by the influx of extracellular calcium and endoplasmic reticulum (ER) Ca^2+^ excretion cause genome disintegration and the subsequent death of a cell by the activation of specific Ca^2+^- and Mg^2+^-dependent endonuclease [210,211,212]. Moreover, Ca ions influence the increased activity of oxidative reactions. Krebs cycle stimulation is dependent on Ca^2+^ concentration in the mitochondria, and the products of this metabolic reaction, FADH2 and NADH, supply electrons to the electron transport chain. In hypoxemic conditions, an elevated number of electrons released in electron transport chain may transfer to molecular oxygen, forming superoxide anions, which are harmful to the surrounding cells [213,214,215]. Both processes, in which Ca plays an important role, may contribute to an increased risk of carcinogenesis. Studies pointed to the significant role of calcium signaling in breast cancer progression. Chamlali et al. reported the store-operated Ca^2+^ entry pathway that is modulated by Ca^2+^ release-activated channel activation. STIM1 is a sensor protein whose conformation changes when the Ca^2+^ stored in the ER is released. Following this, STIM1 opens the plasma membrane’s ORAI channels, which facilitate Ca^2+^ influx and restore the Ca^2+^ reserves in the cells. Elevated ORAI channel numbers are observed more often in groups of women with breast cancer and corresponds with a more aggressive expansion of the tumor. It may be the result of the angiogenesis stimulation caused by Ca^2+^ ORAI1 regulated by Ca^2+^, which plays an important role in breast cancer invasion. Research has shown that high levels of calcium and the dysregulation of signaling processes dependent on Ca^2+^ in women who suffer from breast cancer are associated with an increased rate of deaths [216,217,218].

### 3.11. Magnesium

Magnesium (Mg) functions as a component in a significant number of enzymes that affect a wide range of physiological activities in the body, including translation, signaling processes, and neuronal transmission. It plays an important role in homeostasis by regulating the glucose levels or blood pressure. Moreover, Mg^2+^ might be an important factor in breast cancer development risk due to its influence on homeostatic processes the dysregulation of which may contribute to carcinogenesis [219,220,221]. The recommended daily value for magnesium is 320–420 mg, while its best dietary sources are green leafy vegetables, legumes, nuts, seeds, and whole grains [222]. Any Mg deficiencies might be detected using blood tests. Firstly, Mg is necessary for the proper functioning of nucleic acids, nucleoside triphosphates, deoxyribonucleoside triphosphates, adenosine triphosphate, and peptides by stabilizing their molecular structures. Furthermore, mitochondrial processes and telomeres’ stability are dependent on Mg^2+^ concentrations. The changes in Mg^2+^ concentration can control several activities, such as the regulation of metabolite circulation through glycolysis, the Krebs cycle, glycolysis, oxidative phosphorylation, and adenosine triphosphate release from the mitochondria. Mg^2+^ also affects the lamina, which is a framework consisting of intermediate filaments, responsible for supporting the structural integrity of the nucleus, organizing chromatin, and regulating transcription. By modifying the lamin tail domain, which is recognized by lamin-binding proteins and supplies a place to attach chromatin, Mg^2+^ plays an important role in providing the stability of telomeres [219,223,224,225]. Research has shown that the impaired antioxidant resistance and increase in inflammatory cytokines related to decreased intracellular levels of Mg^2+^ correspond with a higher incidence of carcinogenesis and metastatic spread. Mendes et al. reported that the decreased level of Mg^2+^ causes nitric oxide generation. Nitric oxid triggers the enzymatic production of the proinflammatory interleukin 1 (IL-1) and VEGF, which promote the development of adhesion molecules which cause modifications in cells. Moreover, they have discovered that patients with breast cancer present an increased expression of membrane-bound transporters, specifically TRPM7 and CNNM3, which cooperate to maintain magnesium homeostasis. It has been suggested that the increased number of TRPM7 promotes metastasis onset while CNNM3 may influence breast cancer development by cooperating with the PRL2 oncogene [226,227,228]. There have been reports that, by suppressing the oncogene–Mg^2+^ transporter interaction, it is possible to stop the progression of a tumor [229,230]. Furthermore, research has mentioned a possible relationship between elevated Mg^2+^ levels in urine and breast density that correlates with the risk of breast cancer occurrence [231,232]. It has been reported that magnesium supplementation may increase the overall rate of breast cancer survival [233].

### 3.12. Sodium

A mean target for sodium (Na) daily intake is below 5 g/day; it is contained in foods such as snacks, vegetables, water, and dairy products [234]. In order to assess the body’s Na concentration, either blood or urine tests are performed. Biological cells require Na for proper functioning. Ninety percent of the osmolality of extracellular fluid is composed of Na^+^, the principal cation, and related anions [235,236]. It has been reported that Na^+^ might have a potential influence on breast cancer onset. It has been discovered that increased levels of one of the epithelial sodium channel (ENaC) subunits, the α-ENaC, correlate with the increased expression of E-cadherin (CDH1), a protein whose mutations play an important role in breast cancer development. However, Ware et al. reported a correlation between elevated α-ENaC expression and the onset of a breast cancer subtype that is less aggressive. In their study, the most malignant type of breast cancer, basal-like tumors, presented the lowest expression levels of α-ENaC. In TNBC, a PR-, ER-, HER2- tumor, and one with the worst prognosis, the expression of α-ENaC was low but higher than in basal-like tumors. The highest α-ENaC expression was observed in both PR+ and ER+ breast cancer types which have a better prognosis. Studies have also shown the negative association between α-ENaC expression and Ki67 rate, which is another important factor in tumor grading [237,238,239]. The high levels of Na^+^ contained in processed foods were also taken into consideration as a possible factor in breast cancer development. Research has shown that a high consumption of processed foods correlates with increased incidents of postmenopausal breast cancer onset. However, it has been discovered that weight gain is the most important factor in tumor development. There is poor information about the role of the Na^+^ contained in processed foods in the process of carcinogenesis [240]. Therapeutic products in which Na^+^ is one of the components present promising effects in breast cancer treatment. There are reports showing the prospective use of sodium butyrate combination therapy due to its inhibiting activity on breast cancer cells [241,242,243]. Moreover, Pang et al. suggest the potential role of sodium cantharidate in breast cancer treatment. In animal models, tumor progression was significantly decreased due to the sodium cantharidate intake [244].

### 3.13. Potassium

Potassium (K) is crucial for the physiological properties of cells. The daily intake for potassium is between 2600 and 3400 mg; it is contained in fruits, vegetables, legumes, and dairy products [245]. K deficiencies might be easily evaluated using blood tests. The membrane potential, which affects muscle, nerve, and heart function, is dependent on the K^+^ levels [246,247]. There have been reports that the potassium ions that are dependent on their level might play an important role in breast cancer onset. Lastraioli E. reported on 1.3 voltage-gated potassium ion channels (KV channels), the overexpression of which could correlate with the progression of breast cancer [248]. It has been demonstrated that the KV 1.3 channels are linked to a bad prognosis in patients with breast cancer. Studies have shown that a lower KV 1.3 channel expression is negatively correlated with tumor stage and grade. The use of KV 1.3 channel inhibitors may significantly improve treatment and lead to an increased survival rate among patients with breast cancer [248,249]. Furthermore, Breuer et al. reported another voltage-dependent potassium channel—the KV 11.1 channel—encoded by the KCNH2 gene [250]. It has been discovered that this channel regulates the excitability of membranes, and its low expression is associated with faster breast cancer progression and a worse prognosis. Research has shown that the activation of KV 11.1 channels’ expression inhibits tumor growth and metastasis onset by reversing the epithelial–mesenchymal transition. In this case, the application of the KV11.1 channel’s activator, NS1643, considerably reduced the progression of the tumor [250,251]. Frajese et al. pointed out the potential therapeutic effect of combined potassium–ascorbic acid use in patients with breast cancer. Studies have shown that a potassium–ascorbic acid combination significantly radically reduces the growth of MCF-7 cells in vitro [252].

### 3.14. Phosphorus

Phosphorus’ daily intake should be approximately 700 mg/day; it is contained in protein-rich foods [253]. In order to assess P deficiencies, simple blood tests are performed. Studies show that phosphorus can be used as a non-invasive tool for monitoring cancer therapy, as observing its metabolism illustrates cell energy well. This can be especially seen in breast cancer tissue, which makes this micronutrient possibly useful as a breast cancer biomarker [254]. Phosphorus and its homeostasis are also closely associated with calcium and vitamin D, the low levels of which have been reported in the literature for their frequent presence in patients with breast cancer [8]. This leads us to a greater magnitude of chances for osteoporosis and bone mineral density, whose connection to breast cancer remains ambiguous [255]. The most relevant seems to be the hypothesis that, in breast cancer genesis and progression, significant are not only the levels of different micronutrients and vitamins but also the correlations between them. Furthermore, evidence supports that patients with breast cancer have increased levels of phosphorus in their serum [256]. Its toxicity and dysregulated metabolism may be strongly associated with the genesis of this disease [255], as not only does this study point to a high-Pi environment as a cause of secreted factors capable of increasing both endothelial cell migration and tube formation, leading to angiogenesis, tumor growth, and disease progression [257], but also other studies indicate that even higher dietary phosphorus intakes—which include the Western diet—can lead to tumorigenesis, as they may cause the increased proliferation of cells with malignant transformations [258]. Most of the studies mentioned above emphasize the importance of clinically testing low-phosphate diets’ effects on patients with breast cancer as it may bring important results and new directions in their treatment. In the year 2022, Mendelian randomization was published, which aim was to investigate potential causal associations between genetically predicted circulating concentrations of minerals—including phosphorus—and vitamins and the risk of breast cancer occurrence. This analysis did not confirm an association with breast cancer in total but found an inverse association for diseases with a negative ER status [231]. A very interesting look at this topic is also mentioned in a study from the year 2023, whose aim was to find factors influencing health-related quality of life among patients with breast cancer [259]. The results point out that the serum phosphorus ion levels had a negative effect on the quality of life (QOL) score; however, no study has ever confirmed this connection. All the research mentioned above accurately shows that this direction of studies should be investigated further, as it may lead to significant and helpful results for patients with breast cancer.

### 3.15. Sulfur

Phytochemicals are currently one of the prevalent elements in anticancer drug-based development. A part of them are bioactive organo-sulfur compounds, and they can be mostly found in cruciferous vegetables such as broccoli, cauliflower, or Brussels sprouts. Evidence supports that they can have a significant impact on carcinogenesis through the modulation of DNA methylation and histone modifications, which may result in changes in key suppressor genes, oncogenes, and oncogenic miRNAs [39]. Other studies show that this includes specifically breast cancer. The mentioned mechanisms involve epigenetic changes like the above and also inflammation, angiogenesis, and oxidative stress response [260]. A different study points also to sulfur atoms showing a significant interaction with the human ERα, which may play a crucial role in anticancer activity and has potential as a molecular target. A new direction of research is sulfur compounds—arylofluorosulfates—that specifically inhibit the proliferation of breast cancer cells because of their ability to downregulate the ER mentioned above [261,262]. Also, recent research on diallyl disulfide and diallyl trisulfide suggests that they may suppress malignant breast tumor growth [12,263,264]. The mechanisms of this action are strongly connected to the sulfur compounds collected in a substance called allicin, which can be found in garlic. Studies present that they demonstrate antioxidant and anti-inflammatory effects as well as reduce cytokine secretion and TNF-α. Moreover, they protect cells from oxidative stress reactions and show a cardioprotective activity. Other beneficial aspects of this substance are the stimulation of tissue repair and cardiovascular protection [265]. A different important component is S-allyl-L-cysteine, present in aged garlic extract, which regulates the pathological reactions in cancer cells but also exhibits antihepatotoxic and neuroprotective abilities [266]. Furthermore, a homemade fresh garlic extract has also been predicted to show anticancer activity, as it reduced tumor growth in preclinical mouse models with breast cancer [267]. Furthermore, a study from 2022 pointed to a low-sulfur amino acid diet as another possible inhibitor of breast cancer growth as it leads to decreased angiogenesis within the tumor mass as well as lower immune cell infiltrates and inflammatory cytokine production [268]. A lot of studies also focus on the iron–sulfur (Fe-S) clusters that form a protein, the nutrient deprivation autophagy factor-1, which is correlated with different cancer types [269,270,271,272]. The above-mentioned factor has a significant impact on redox reactions, DNA replication, and telomere maintenance. Evidence also supports that the iron–sulfur cluster’s metabolism is correlated with cellular resistance to oxidative stress, which has an important impact on cell proliferation and disease progression. This leads to a new direction of research on possible pharmaceutical inventions that will inhibit the accumulation of the nutrient deprivation autophagy factor-1 or secure its stability. However, this topic still remains not fully tested, and studies emphasize the significance of further research on iron–sulfur proteins. All the above support the hypothesis of sulfur not only being one of the natural inhibitors of this disease but also a modifying factor of each tumorigenesis stage and a possible new therapeutic method for patients with drug resistance.

### 3.16. Fluorine

A daily intake of fluorine around 3–4 mg is mostly satisfied by tea and coffee, seafood, and fortified water, as well as dairy products [273]. Fluorine is one of the main elements used in breast cancer diagnosis as it can be found in a substance called 18-fluorodeoxyglucose, which is a very optimal tool for imaging the glucose metabolism in cells. The most frequently used is ^18^F-FDG PET/CT, which helps, among other things, with the identification, staging, and evaluation of therapy response and with the recurrence monitoring of breast cancer [274,275,276,277,278]. Fluorine atoms are small and electronegative, which allows them to increase the lipophilicity of a created compound. Furthermore, there are other advantages to this element, such as drug–target interactions, specificity, metabolic stability, acidity or basicity, membrane permeability, and toxicity. On top of this, nowadays, fluorine commonly takes part in the development of drugs for many different diseases, and its contribution to medicinal chemistry seems to be important as new fluorine-containing compounds are explored. A very good example is isoflavones, which showed increased biological activity against cancer cells after being fluorinated [279]. Moreover, a recent study from 2022 showed that the strategic incorporation of fluorine and organofluorine groups to new-generation taxoid anticancer agents, acylhydrazone-based antifungal agents, and matrix metalloproteinase 9 (MMP-9) can better the effects of these substances in therapies and presents a hopeful direction for further research [280]. Also, another recent study, which focused on fluorine-incorporated gold compounds, states its possible anticancer activity. The authors used TNBC cells, ER-positive breast cancer cells, and normal myoblast cells to test for growth inhibition, apoptosis induction, and cell cycle arrest. The conclusions are promising, as this substance showed a positive effect in all those mechanisms and the potential to become a new anticancer drug [281]. Furthermore, another substance called thiazolylhydrazone expressed a significant activity against cancer cells after it was introduced with fluorine. Scientists tested its impact on TNBC cells, and the results showed the inhibition of proliferation, migration, and invasion. Furthermore, fluoro–thiazolylhydrazone promoted the apoptotic rate by the mitochondria apoptosis pathway [282]. Considering the above-mentioned articles, fluorine’s anticancer activity potential still remains not fully discovered, and its compounds are worthy of further analyses and are a hopeful direction for research on a cure against breast cancer progression.

### 3.17. Iodine

Iodine is strongly connected to the thyroid, as it is part of its hormones, and the prevalent amount of this element is stored exactly in this organ. Smaller amounts can be found in our stomach, breast, or skin. A daily intake of iodine around 150 µg is mostly satisfied by food rich in iodine such as fish, crustaceans of marine origin, seaweeds, and sea vegetables [283]. In order to assess the body’s iodine levels, either blood or urine tests can be performed. In some European countries, not all the above-mentioned products are very popular in a diet, and that is why locals often regulate their iodine levels with salt rich in potassium iodine (KI) [284]. The sufficiency of iodine in our bodies can be measured with urinary iodine excretion (UI), as most of it is eliminated with this fluid, and it has been stated by the World Health Organization that results below 100 µg/L equal an iodine deficiency. The literature supports the fact that low levels of this element are associated with an increased risk of cancer, especially thyroid cancer; however, studies show that it can be also connected with other types of malignant tumors such as stomach cancer, lung cancer, or breast cancer [285]. A prospective pilot study showed results regarding the correlation between UI and breast cancer. A total of 24 patients with 48 controls were examined for their urine iodine concentration; the results showed no statistically important differences between the two groups; however, a higher percentage of patients had an index above 200 µg/L [286]. Although this suggests a better role of iodine as a marker, other studies support the usage of this element as a very effective treatment method for breast cancer as it presents apoptotic, angiogenesis, and antiproliferative activity. A recent study examined the impact of molecular iodine on the immune component in mammary tumors. The patients were receiving a chemotherapy treatment and I2 or a placebo after breakfast in a double-blind system. The conclusions are that iodine supplementation induced the activation of the immune system, promoted an antitumor response (Th1), and increased the cytotoxicity of intratumoral NK and CD8+ cells, and, thus, the patients receiving a combined therapy had the best results in their treatment, such as a smaller tumor size and the cancellation of chemo resistance [287]. Very similar results had been observed in some previous studies [288]. Moreover, the literature supports that iodine can also be used in photoimmunotherapy [289] and brachytherapy [290,291] and has potential in targeted radionuclide therapy [292]. A very interesting application of iodine is in 125I seeds, which can be implanted into a patient’s lesion to provide treatment, improve the treatment’s effects, reduce adverse reactions, and better the quality of life. Significant results of this method were shown in a study in which two groups of 45 patients with breast cancer bone metastases were compared after one of them received conventional therapy and the other a treatment of radioactive seed 125I implantation under CT guidance. The effective rate of the experimental group was statistically higher, which showed the positive effects of iodine usage in therapy and emphasized the importance of further research on this topic [293]. Furthermore, studies support that 125I seeds are safe to use for pregnant women with breast cancer as a localization technique [294]. Studies describing the involvement of the aforementioned trace elements in the onset and progression of breast cancer are presented in Table 2.

## 4. Conclusions

Micronutrient status seems to play a significant role in breast carcinogenesis as well as the progression of disease symptoms. In this narrative review, we summarized the current knowledge about 23 different minerals and vitamins, taking into account their role in maintaining homeostasis as well as the results which appear to be due to their imbalanced concentrations, such as the increased risk of breast cancer or the unsatisfactory results of cancer therapies. Knowledge regarding the proper doses of micronutrient supplementation that seem to be satisfactory in preventing carcinogenesis or alleviating the symptoms of cancer might be crucial in providing proper and effective therapeutic approaches as a holistic approach to breast cancer treatment. Modulating the internal processes of cancer cells via the mechanisms of action of chosen micronutrients can potentially inhibit the growth of the tumor. Different biological features of the described nutrients, such as antioxidative features, the modulation of different enzymes’ activity, or combined therapy, in which macro- and micronutrients are crucial aspects, provide a promising effect in breast cancer regression and recovery. However, further research on this matter is highly recommended because of the huge number of contradictory results in the evaluated studies. Further, since knowledge regarding micronutrient status and the resulting possible risk in terms of the onset and/or progression of breast cancer is highly limited, it would be favorable to focus on people’s nutritional status throughout their whole life course, taking into account critical periods such as childhood, early adulthood, or the period before cancer onset as well as during therapy. During the whole therapeutic and diagnostic process, clinicians should focus on decisions regarding the intake of specific micronutrients, the deficiencies of which might be associated with further carcinogenesis. Similarly, it should be considered whether specific micronutrients’ overaccumulation could also disturb therapy and alleviate disease symptoms. Further studies should also focus on indicating the patients who constitute a high-risk group based on the available nutritional status of those patients. Such actions could significantly improve both the diagnostic and therapeutic processes.

## Figures and Tables

**Table 1:** Studies describe the association of vitamins with breast cancer.

Authors	Year	Origin	Number of Patients	Vitamin	Results
Jain et al. [27]	1994	Canada	89,835 women (1270 histologically confirmed cases of invasive carcinoma of the breast)	Vitamin A	Association between pre-diagnosis dietary β-carotene intake and prognosis in patients with breast cancer with estrogen receptor (ER+) or (PR+) status, but not in those with ER- or PR-negative status.
Sutton et al. [30]	1997	United States	17 women with metastatic breast cancer	Vitamin A	The recommended daily dose of 150 mg/m^2^ ATRA for 14 consecutive days did not demonstrate significant activity in breast cancer.
Eliassen et al. [26]	2015	United Kingdom	1976: 121,701 women 1989–1990: 32,826 women 2000–2002: 18,743 2010: 32,826	Vitamin A	High levels of plasma carotenoids are associated with a 28% lower risk of breast cancer in a 20-year follow-up.
Kim et al. [48]	2019	Canada	129 women with breast cancer	Vitamin B	Moderate folic acid- and vitamin B12-containing supplement use may be protective for BRCA-associated breast cancer.
Harris et al. [83]	2013	Sweden	3405 participants with invasive breast cancer	Vitamin C	Dietary intake before breast cancer diagnosis may be associated with breast cancer survival. Post-diagnosis supplementation was not associated with survival.
Manson et al. [102]	2019	United States	25,871 participants; cancer was diagnosed in 1617 participants (793 in the vitamin D group and 824 in the placebo group)	Vitamin D	Supplementation with vitamin D did not result in a lower incidence of invasive cancer.

**Table 2:** Studies describe the association of minerals with breast cancer.

Authors	Year	Origin	Number of Patients	Trace Element	Results
Chan et al. [133]	2017	United States	75 women with breast cancer	Copper	The efficacy of the oral copper chelator tetrathiomolybdate was confirmed. The OS was 84% with a median follow-up of 6.3 years.
Barartabar et al. [122]	2023	Iran	40 women with a histologically confirmed diagnosis of invasive ductal carcinoma	Copper	The copper levels in the tumor tissue were significantly higher than in the tumor margin tissue.
Liu et al. [146]	2019	China	229 women with breast cancer (167 cases of primary invasive breast cancer and 62 cases of lymph node metastatic breast cancer)	Cobalt	The overexpression of cell cycle-related proteins (p38MAPK, ERK, JNK, and CDC25C) can promote PGCC formation to facilitate invasion and metastasis in breast cancer, worsening patient prognosis.
Sahin et al. [158]	2022	Iran	Randomized group of 181 women with breast cancer and a placebo group of women	Boron	The efficacy of a boron-based gel in preventing acute dermatitis, erythema, dry desquamation, and moist desquamation in patients undergoing radiation therapy for breast cancer was confirmed.
Jablonska et al. [166]	2017	Poland	42 female patients with breast cancer	Selenium	The dysregulation of Se homeostasis and the accumulation of cadmium in tissues are associated with the development of breast cancer. Breast cancer metastases are most often localized in tissues with a low Se content.
Guo et al. [165]	2020	United States	9487 female patients with breast cancer	Selenium	No association between the total Se levels, the dietary Se content, or Se supplementation and breast cancer incidence in postmenopausal women.
Sandsveden et al. [164]	2020	Sweden	1066 female patients with breast cancer	Selenium	The lower mortality rate in women with high serum Se levels at the moment of diagnosis was confirmed.
Szwiec et al. [163]	2021	Poland	538 female patients with breast cancer	Selenium	Low serum Se levels at the moment of diagnosis were associated with an increased risk of death over the next 10 years in female patients with breast cancer.
Shen et al. [173]	2015	China, Turkey, Korea	1302 participants	Manganese	A significant association between deficient Mn levels and breast cancer.
Liu et al. [204]	2021	China	1591 patients with breast cancer	Iron	Significant non-linear J-shaped associations between total dietary Fe and breast cancer risk.
Huang et al. [227]	2019	China	1050 case participants	Magnesium	A direct negative association and an indirect association through influencing the CRP level were observed between dietary magnesium intake and breast cancer risk.
Chen et al. [259]	2023	China	246 patients with breast cancer	Phosphorus	The serum phosphorus ion level had a negative effect on the QOL score.
Malya et al. [286]	2018	Turkey	24 female patients with breast cancer and 48 controls	Iodine	No statistically important differences between the two groups in iodine excretion; however, a higher percentage of patients had an index above 200 µg/L.
Cuenca-Micó et al. [287]	2021	Mexico	30 patients with breast cancer receiving a chemotherapy treatment with molecular iodine or a placebo in a double-blind system	Iodine	Iodine supplementation induces the activation of the immune system. Patients receiving a combined therapy had the best results in their treatment, such as a smaller tumor size and the cancellation of chemo resistance.
Li et al. [293]	2022	China	90 patients with breast cancer bone metastases split into a control group and an experimental group, with 45 cases in each group.	Iodine	The effective rate of the experimental group with the treatment of radioactive seed ^125^I implantation under CT guidance was statistically higher than in the control group with conventional therapy.

QOL—quality of life.

## Data Availability

Not applicable.

## Abbreviations

ATRA	all-trans retinoic acid
BNCT	boron neutron capture therapy
BON	biodegradable boron oxynitride
BPA	boronophenylalanine
BT	barium titanate
CA IX	carbonic anhydrase
CoCl_2_	cobalt chloride
CRBP-1	cellular retinol binding protein-1
CRGs	cuproptosis-related genes
DCs	dendritic cells
DHA	dehydroascorbic acid
EMT	epithelial–mesenchymal transition
ENaC	epithelial sodium channel
ER	estrogen receptor
ER	endoplasmic reticulum
GLUT1	glucose transporters 1
H_2_O_2_	hydrogen peroxide
HER2	human epidermal growth factor-2
HIF-1α	hypoxia-inducible factor
IL-18	interleukin 18
KV channels	potassium ion channels
LIPT1	lipoyltransferase 1
MMP-9	matrix metalloproteinase 9
MMPs	matrix metalloproteinases
MnSOD	manganese superoxide dismutase
MoS_2_	molybdenum disulfide
PGCCs	polyploid giant cancer cells
PR	progesterone receptor
RA	retinoic acid
RARs	retinoic acid receptors
ROS	reactive oxygen species
RXRs	retinoid X receptors
TNBC	triple-negative breast cancer
TNF-α	tumor necrosis factor α
VEGF	vascular endothelial growth factor
VK2	vitamin K2
VK3	vitamin K3
ZIP6	Zrt-Irt-like protein 6
